# Nothing about us without us: involving patient collaborators for machine learning applications in rheumatology

**DOI:** 10.1136/annrheumdis-2021-220454

**Published:** 2021-07-05

**Authors:** Stephanie J W Shoop-Worrall, Katherine Cresswell, Imogen Bolger, Beth Dillon, Kimme L Hyrich, Nophar Geifman, Members of the CLUSTER consortium, Elizabeth Ralph

**Affiliations:** 1 Centre for Health Informatics, The University of Manchester, Manchester, UK; 2 Centre for Epidemiology Versus Arthritis, The University of Manchester, Manchester, UK; 3 NIHR Manchester BRC, Manchester Academic Health Science Centre, Manchester University NHS Foundation Trust, Manchester, UK; 4 Vocal, Manchester University NHS Foundation Trust, Manchester, UK; 5 Your Rheum, Young Person’s Research Advisory Group, Manchester, UK; 6 Faculty of Health and Medical Sciences, University of Surrey, Guildford, Surrey, UK

**Keywords:** arthritis, juvenile, epidemiology, outcome assessment, outcome and process assessment, health care, patient reported outcome measures

## Abstract

Novel machine learning methods open the door to advances in rheumatology through application to complex, high-dimensional data, otherwise difficult to analyse. Results from such efforts could provide better classification of disease, decision support for therapy selection, and automated interpretation of clinical images. Nevertheless, such data-driven approaches could potentially model noise, or miss true clinical phenomena. One proposed solution to ensure clinically meaningful machine learning models is to involve primary stakeholders in their development and interpretation. Including patient and health care professionals’ input and priorities, in combination with statistical fit measures, allows for any resulting models to be well fit, meaningful, and fit for practice in the wider rheumatological community. Here we describe outputs from workshops that involved healthcare professionals, and young people from the Your Rheum Young Person’s Advisory Group, in the development of complex machine learning models. These were developed to better describe trajectory of early juvenile idiopathic arthritis disease, as part of the CLUSTER consortium. We further provide key instructions for reproducibility of this process. Involving people living with, and managing, a disease investigated using machine learning techniques, is feasible, impactful and empowering for all those involved.

## Unsupervised machine learning in healthcare

Recent years have seen a rapid growth in artificial intelligence applications, such as machine learning, to healthcare^1^ allowing for the prediction of outcomes and identification of patterns within increasingly complex datasets. Therefore, applications such as automated interpretation of X-ray or MRI, decision support for therapy selection and data-driven classification of heterogeneous conditions may become common practice.^2^

In rheumatology, these approaches could help better define and map outcomes in patients with complex diseases such as juvenile idiopathic arthritis (JIA) or systemic lupus erythematosus. While supervised machine learning applications are trained to classify or make predictions for patients, unsupervised machine learning methods allow for data-driven pattern detection, or clustering, of people without using predefined clinical criteria.^1 2^ These clusters may represent those with unique disease features at a single clinic visit or with distinct disease trajectories over time. Although each person with a disease is unique and the entirety of their disease impact should be considered when providing treatment, guidelines for treatment are developed for mass application and rely on population-based criteria. Identifying similar experiences within groups of people can allow for tailoring of therapies and forecasting of disease course in a more pragmatic paradigm that can be applied to treatment guidelines. In addition, people within groups with similar disease manifestations or experiences may have separate clinical and biological mechanisms that underpin their data-driven clusters, for example, following specific antirheumatic therapies.^3 4^ Data-driven clustering methods are, therefore, a potential gateway to stratified medicine across rheumatology.

Clusters identified through unsupervised machine learning methods may prove to be more clinically relevant than those defined by preset clinical criteria; participants are grouped using factors that may be crucial in terms of outcome but not evident to clinicians; however, their flexibility means that modelling of noise within a dataset, which does not represent true variation between patients or disease courses in clinical practice, is a possibility.^2^ Furthermore, a lack of a ground truth means that researchers are faced with several potential ‘optimal’ models to choose from, and validation of any resulting groupings is not straightforward. Current machine learning paradigms suggest that final model selection be driven through optimising parsimony without compromising model fit, for example, selecting the model at the ‘elbow’ of a Bayesian Information Criterion (BIC) curve.^5 6^ However, the selection of a more parsimonious model might miss a true clinical phenomenon, while a more complex, better-fitting model may inadvertently overfit the data, modelling data quirk rather than clinically meaningful differences. Both of these scenarios result in the potential for suboptimal patient care where subgroups with unique disease features are missed or subgroups that do not exist clinically are incorrectly treated differently due to these analytical constraints for current model selection.

## Why involve people with the disease and medical practitioners in machine learning research?

One proposed solution to improve selection of clinically meaningful models is to include primary stakeholders in the construction of machine learning applications. People living with a given disease have unique viewpoints, allowing for research to be directed and refined based on first-hand experiences. Excluding these people from the research process means that both their agency and their priorities are likely overlooked.^7^ Healthcare professionals play a crucial role in the management of disease in these primary stakeholders; their priorities often differ from people directly affected by disease^8^ but should also be reflected in machine learning models built for healthcare.

### Key instruction 1

Involve people who live with and those who treat diseases in research about their condition of interest.

### An example: finding clusters of children and young people (CYP) with JIA

JIA is the most common inflammatory arthritis of childhood. It is a heterogenous condition and approach to, as well as response to, treatment is not universal, with a significant proportion of children known to have persistent disease, chronic symptoms and associated comorbidity^9 10^ despite treatment. Through the CLUSTER consortium (www.clusterconsortium.org.uk), as part of our efforts to improve personalised treatment in JIA, we aimed to identify clusters of CYP who experience distinct patterns of arthritis-related outcomes.^11^ These outcomes were assessed following diagnosis based on clinical data captured within the Childhood Arthritis Prospective Study, a UK multicentre inception cohort of JIA. Using group-based trajectory models, a form of unsupervised machine learning, we clustered approximately 1200 CYP, based on their recorded number of affected joints, a physician global assessment of their disease activity and a patient global assessment of their well-being over time.^12^

The initial results from the clustering analysis revealed a shortlist of models that all fit criteria for good model adequacy, fit and discrimination between identified clusters.^6^ These models were brought forward for discussion with key stakeholders through structured workshops.

### Key instruction 2

Present and discuss with involvement groups only well-fitting models, to ensure final results both well describe the data captured and are clinically meaningful.

### Patient and healthcare professional involvement in model selection

**Figure 1:**
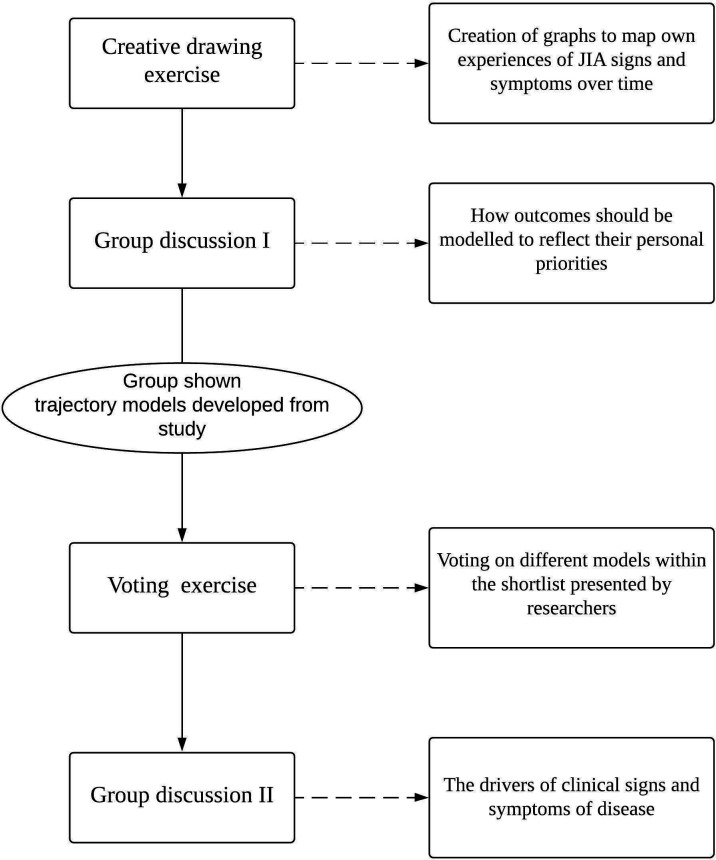
The process by which machine learning models were ratified through patient and healthcare professional involvement. JIA, juvenile idiopathic arthritis.

Potential models were discussed in separate focus groups with CYP and healthcare professionals. The CYP group included members of the young person’s advisory group Your Rheum,^13 14^ consisting of CYP aged 11–24 years with musculoskeletal conditions across the UK. Seven of these members (aged 14–22 years) were involved in the current study. The healthcare professional group consisted of 12 multidisciplinary rheumatology specialists (paediatric rheumatology, physiotherapy, occupational health, nursing, research practitioner, trainees) within Royal Manchester Children’s Hospital at Manchester Foundation NHS Trust. These groups were consulted with the specific aims of identifying the most clinically relevant models that represent real-world experiences of CYP and healthcare professionals, but avoiding modelling of noise, from our shortlist of well-fitting models. Both groups undertook four activities supervised by the researcher, to initially ground them in interpretation of trajectory plots through drawing their own experiences, to aid in outcome selection through a group discussion and then to select and discuss models most relevant to their experiences (figure 1).

### Key instruction 3

Include an educational and interactive activity before discussing complex research, to ground the involvement groups in the methods, bring key experiences to the forefront of their minds and facilitate initial discussion.

### Choosing outcomes for machine learning studies

Core outcomes in JIA represent patient-reported and physician-assessed variables which may hold different levels of importance to people with the disease and healthcare professionals, respectively. Machine learning research should consider consulting involvement groups to select and include outcomes relevant to both parties.

**Figure 2:**
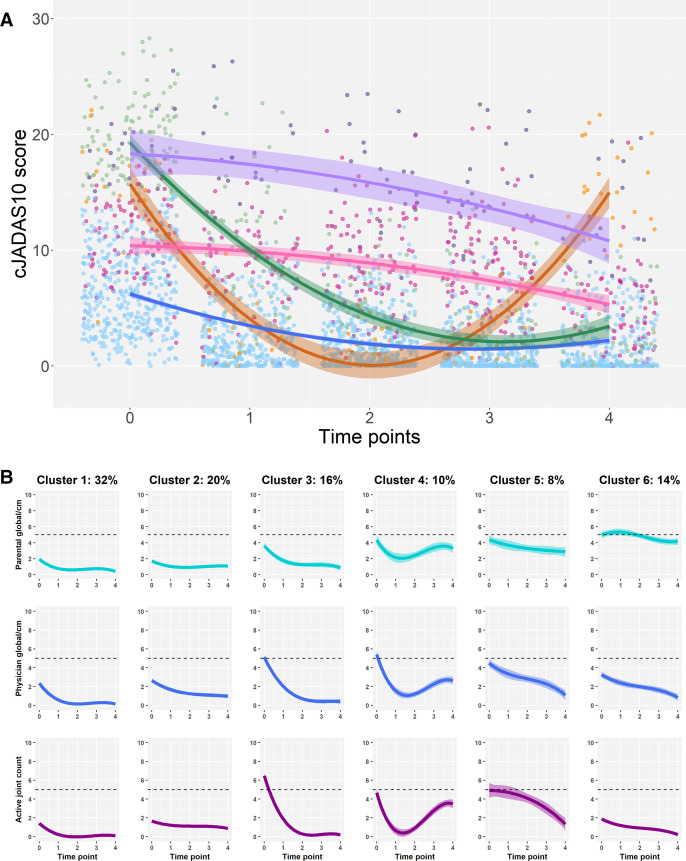
(A) Univariate and (B) Multivariate modelling approaches presented as alternatives to the focus groups, adapted from Shoop-Worrall *et al*.^12^ Both groups considered multivariate modelling (B) more meaningful. Each trajectory represents an average outcome pattern for one cluster of CYP with JIA. For all outcomes, higher scores denote more severe outcomes. (A) Five clusters of CYP, each with a different average pattern of the cJADAS10 score over time. (B) Six overall clusters, each with unique shared patterns of parental global scores, physician global scores and active joint counts over time. CYP, children and young people; JIA, juvenile idiopathic arthritis.

The models presented to the focus groups clustered CYP with JIA based on changes across multiple core outcomes, which can be combined into a composite outcome, producing a single score to represent overall disease impact.^15^ Both groups suggested that their experiences or treatment decisions would hinge on specific outcomes within those included in the models, thus bringing into question the utility of a composite score for research assessing clusters of disease. For young people, clusters identified by modelling the outcomes separately rather than using the composite score were deemed more meaningful (figure 2). For this group, separating changes in well-being from physician-assessed measures was key to understanding how they would experience their disease over time. Young people were particularly concerned that the research should demonstrate separate trajectories for physician global scores and patient well-being scores, since these measure different aspects of disease impact and many young people had experienced physician-assessed disease activity and self-perceived well-being not aligning with one another.

For healthcare professionals, modelling the outcomes separately rather than as a composite score was also important (figure 2); modelling all outcomes as a composite score would not delineate changes in joint count from changes in more subjective measures on which they would be less likely to base antirheumatic drug or physiotherapeutic decisions.

Based on discussions with both groups, multivariate modelling was therefore preferred to allow the identification of clusters with unique characteristics across outcomes prioritised by different key stakeholder groups who may have different goals of treatment.

### Key instruction 4

Involve key stakeholders in selecting outcomes of research to best fit their priorities. Note that different groups of stakeholders may have different priorities, and efforts should be made to facilitate each of these.

### Prioritising clinically meaningful models while minimising noise

Distinguishing features between competing, well-performing, models included the addition or removal of a cluster or differences in polynomial structure, or pattern of change, observed over time. For the young person’s group, the most complex model (cubic polynomial) showed clusters with distinct, meaningful patterns of disease and well-being, even though this model would not have been objectively selected using the elbow approach. An additional cluster was depicted by this model (cluster 2, figure 2B) and the researchers were unsure of clinically meaningful difference to an existing cluster (cluster 1, figure 2B). The group suggested that this additional cluster represented a unique experience of disease over time. However, healthcare professionals noted that the profile of this new cluster would only change their treatment decisions compared with a more parsimonious model if the magnitude of change was demonstrated in the active joint count outcome. Had a similar difference between clusters been observed in one of the other outcomes, it would not have influenced their treatment decisions, particularly for the paediatric rheumatologists considering antirheumatic drug therapy. This exemplifies the utility of presenting results to CYP and healthcare professionals, where better fitting, more complex models may not identify clinically distinct groups of patients in terms of the patient experience or management of disease in its current form. In these cases, more parsimonious models with less optimal fit may be more clinically useful and/or better describe the patient experience; however, in this instance, the most complex model was deemed to cluster young people based on meaningful differences in disease, rather than noise in the dataset, and was therefore selected.

### Key instruction 5

Be prepared to balance clinical relevance and statistical fit. The objectively ‘best-fitting’ model may be noisy or overfit, and stakeholders can help identify when a more parsimonious model would be more clinically helpful.

### Feasibility of involving primary stakeholders in machine learning research

Involving young people in interpreting research, particularly when asking them to recall their own disease journey, is a very personal experience and therefore requires a greater level of sensitivity than involvement for planning or disseminating research. Potential barriers to effective involvement with these young people were perceived to be the potentially sensitive nature of recall alongside the complexity of the models presented. Written and verbal feedback on the event from stakeholders was sought to evaluate the experiences of being involved, including difficulty, comfort and enjoyment of the exercises, and suggestions for future events (box 1).

Box 1 **Experiences completing the creative drawing and viewing printed model tasks.**

**(a) Difficulty:**

- (The exercise was) difficult to begin with as there were things you had to decide, such as ‘what is my timescale?’ and ‘what classes as good or bad?’
- Seeing other people do it made it easier.
- I really enjoyed the drawing.

**(b) Recalling past events:**

- Remembering important events was much easier to do as they are things that stick in your mind.
- I can remember the pattern of illness but can’t remember what it felt like at age 2.
- It might have been easier to do if you were years away from diagnosis as you can look back…in the years closest to you, you almost remember too much.

**(c) Potential for distress:**

- (Looking at the graphs was) personally not distressing, more distressing was putting on the life events as they’re reminders of upsetting times…it was refreshing to be fair.
- Some things may feel close to the bone.
- These are things you don’t often get the chance to talk about, you hide them away.
- Even if it is upsetting, you are doing something useful with it. I find thinking about the future harder.

**(d) Running the sessions:**

- It helps having the right person run the session, somebody who wants to listen.
- I didn’t feel at all forced into doing it. You choose what you want to put down, no-one is inside your head.
- Just make it clear that you don’t have to put anything down you don’t want to.

**(e) Creative drawing as a means of understanding multitrajectory graphs presented:**

- This helped engage with the graphs as you understand it.
- If you faced me with the graphs (without the creative drawing) I wouldn’t have known where to begin.
- Drawing and the arts are really helpful…anything that puts it into perspective helps.
- Especially helpful if you are working with younger kids.
- I love a graph! It is not loads of things to read in complex language.
- I hate graphs but I found it quite enjoyable.

**(f) Overall takeaways from being involved:**

- Getting people talking about it helps you realise that you are not alone.
- The transparency of what (the research) will be used for and how it will help was good.
- It was refreshing to hear the purpose of the research, why we are doing it differently.
- (The research was) really, really worthwhile looking at.

### Key instruction 6

Seek feedback on all involvement activities. This evaluation will improve future efforts for both stakeholders and the overall research.

Despite no previous education on machine learning, the creative drawing exercise successfully familiarised the young people and healthcare professionals with trajectory graphs and the concept of multivariate modelling. Completing the tasks in a group setting appeared to facilitate understanding in addition to fostering reassurance among young people about shared experiences. Young people also felt that recalling past events could be distressing and to always clarify that participants could draw whatever they feel comfortable sharing, with suggestion that some distressing events may be cathartic to discuss in a sensitive and supportive environment (box 1). Young people over the age of 16 years provided written consent to taking part in the involvement group. Parents of young people under the age of 16 years signed consent forms and accompanied their children to the event but were not present for the duration of the meeting. Previous experience with this advisory group has suggested more open conversations and a greater sense of peer support and when young people are able to participate independently.

### Key instruction 7

Plan to facilitate sharing of experiences while minimising discomfort. Peer support is often helpful for both of these and so group sessions may be preferred to one-on-one sessions. Have a distress protocol in place for managing participants’ potential distress.

## Conclusions

Unsupervised machine learning approaches may be the key to unlocking stratified medicine across diseases, including in rheumatology. Involving people with first-hand experience of disease and healthcare professionals who treat it, in key methodological and interpretive decisions, including outcome selection, model selection and model interpretation, can significantly improve unsupervised machine learning based research. Involvement in this type of research is feasible even with young people through creative tasks. Once well-fitting models have been identified using mathematical measures, researchers should consider that their own second-hand or third-hand knowledge of a disease is insufficient to choose a final model. Leveraging experiences from these groups ensures that models produced are: (1) useful to key stakeholders, (2) do not exclude clinically meaningful outputs and (3) minimise identification of noise as a clinical finding. These insights can only be gained through discussions with those closest to the disease.
